# Genomic Epidemiology and Evolution of *Escherichia coli* in Wild Animals in Mexico

**DOI:** 10.1128/mSphere.00738-20

**Published:** 2021-01-06

**Authors:** Robert Murphy, Martin Palm, Ville Mustonen, Jonas Warringer, Anne Farewell, Leopold Parts, Danesh Moradigaravand

**Affiliations:** a University of Copenhagen, Department of Biology, Section for Ecology and Evolution, Copenhagen, Denmark; b Center for Computational Biology, Institute of Cancer and Genomic Sciences, University of Birmingham, Birmingham, United Kingdom; c Department for Chemistry and Molecular Biology, University of Gothenburg, Gothenburg, Sweden; d Centre for Antibiotic Resistance Research at the University of Gothenburg, Gothenburg, Sweden; e Organismal and Evolutionary Biology Research Programme, Department of Computer Science, Institute of Biotechnology, University of Helsinki, Helsinki, Finland; f Helsinki Institute for Information Technology, Helsinki, Finland; g Wellcome Sanger Institute, Wellcome Genome Campus, Hinxton, Cambridgeshire, United Kingdom; h Department of Computer Science, University of Tartu, Tartu, Estonia; University of California, Davis

**Keywords:** *Escherichia coli*, genomic epidemiology, host-pathogen interaction, infectious diseases, whole-genome sequencing, wild animals

## Abstract

Escherichia coli is a common bacterial species in the gastrointestinal tracts of warm-blooded animals and humans. Pathogenicity and antimicrobial resistance in E. coli may emerge via host switching from animal reservoirs. Despite its potential clinical importance, knowledge of the population structure of commensal E. coli within wild hosts and the epidemiological links between E. coli in nonhuman hosts and E. coli in humans is still scarce. In this study, we analyzed the whole-genome sequencing data of a collection of 119 commensal E. coli strains recovered from the guts of 55 mammal and bird species in Mexico and Venezuela in the 1990s. We observed low concordance between the population structures of E. coli isolates colonizing wild animals and the phylogeny, taxonomy, and ecological and physiological attributes of the host species, with distantly related E. coli strains often colonizing the same or similar host species and distantly related host species often hosting closely related E. coli strains. We found no evidence for recent transmission of E. coli genomes from wild animals to either domesticated animals or humans. However, multiple livestock- and human-related virulence factor genes were present in E. coli of wild animals, including virulence factors characteristic of Shiga toxin-producing E. coli (STEC) and atypical enteropathogenic E. coli (aEPEC), where several isolates from wild hosts harbored the locus of enterocyte effacement (LEE) pathogenicity island. Moreover, E. coli isolates from wild animal hosts often harbored known antibiotic resistance determinants, including those against ciprofloxacin, aminoglycosides, tetracyclines, and beta-lactams, with some determinants present in multiple, distantly related E. coli lineages colonizing very different host animals. We conclude that genome pools of E. coli colonizing the guts of wild animals and humans share virulence and antibiotic resistance genes, underscoring the idea that wild animals could serve as reservoirs for E. coli pathogenicity in human and livestock infections.

**IMPORTANCE** Escherichia coli is a clinically important bacterial species implicated in human- and livestock-associated infections worldwide. The bacterium is known to reside in the guts of humans, livestock, and wild animals. Although wild animals are recognized as potential reservoirs for pathogenic E. coli strains, the knowledge of the population structure of E. coli in wild hosts is still scarce. In this study, we used fine resolution of whole-genome sequencing to provide novel insights into the evolution of E. coli genomes from a small yet diverse collection of strains recovered within a broad range of wild animal species (including mammals and birds), the coevolution of E. coli strains with their hosts, and the genetics of pathogenicity of E. coli strains in wild hosts in Mexico. Our results provide evidence for the clinical importance of wild animals as reservoirs for pathogenic strains and highlight the need to include nonhuman hosts in the surveillance programs for E. coli infections.

## Abstract (precis)

Escherichia coli is a clinically important bacterial species implicated in human- and livestock-associated infections worldwide. The bacterium is known to reside in the guts of humans, livestock, and wild animals.

## INTRODUCTION

Escherichia coli is the most prevalent aerobic bacterial species that resides in the intestines and feces of warm-blooded animals and dominates the corresponding microbiomes (1). Hosts provide the bacterium with a constant supply of nutrients and protection against environmental stresses, and the commensal nature of E. coli may facilitate its dissemination across hosts (2, 3). Pathogenic and antimicrobial-resistant (AMR) clones of E. coli have spread rapidly over recent years, and understanding the ecological origins of these strains has therefore become increasingly important. A significant number of acute E. coli infections are known to have zoonotic origins (2). Because half of the total natural E. coli population is estimated to inhabit environmental sites, nonhuman hosts and settings are large potential reservoirs for pathogenic and AMR strains and genes (4).

Despite its likely importance for human health, the genetic diversity of commensal E. coli within wild hosts is not well understood, primarily due to the difficulty of recovering samples. Some studies have suggested that E. coli and colonized hosts coevolve, such that the genomic characteristics of E. coli depend on the host species (5, 6). While neutral evolutionary forces such as genetic drift likely dictate most of the E. coli genetic diversity, microenvironments of gastrointestinal tracts of the hosts may exert powerful selection pressures that influence E. coli feeding habits and diet and contribute to the phenotypic differentiation of commensal strains. These factors have led to E. coli strains from wild animals often falling into other genetic and phenetic clades, and thus into other phylogroups, than isolates retrieved from humans (5–8).

Reports have described high multidrug resistance in individual environmentally sourced E. coli isolates, but strains found in wild animals generally display lower AMR than those found in livestock and nonanimal environmental samples. Further, proximity of wildlife to human settlements seems to influence the AMR of gut microbiomes in wild hosts, likely due to the closely associated antibiotic pollution of land and water environments (9, 10). Interactions between humans and livestock have also been reported to catalyze the colonization of wild-life by AMR E. coli in Nairobi, Kenya. But whether wild animals predominantly act as sources or sinks in AMR evolution is still unclear (11, 12). The distribution of genes encoding virulence factors in environmental E. coli isolates is still an understudied area, although accumulating evidence shows high genetic relatedness between pathogenic strains infecting livestock and those infecting humans. This suggests that jumps between animal and human hosts do occur at epidemiological time scales (13, 14). Furthermore, a recent study on human and environmental strains in Australia found a dominant role for horizontal gene transfer in spreading virulence factor and AMR genes across hosts and showed that host phylogeny and habitat can shape the E. coli genetic diversification (8).

Exploration of the potential environmental, and more specifically zoonotic, origins of AMR and pathogenic E. coli strains and genes requires studies of genetically different bacterial isolates from a wide diversity of sources and geographical regions. Here, we examined the whole-genome sequences of 119 commensal E. coli isolates recovered from the fecal samples of 55 wild mammal and bird species from North America, predominantly from Mexico (5). With an estimated 2,000 different resident mammal and bird species, Mexico hosts 10 to 12% of the corresponding worldwide diversity (15). This allowed us to scrutinize the host-pathogen evolution across a wide range of wild host populations, at a regional level.

Our results indicate that E. coli populations in wild hosts are only weakly associated with the taxonomy and ecological and physiological attributes of the host species. Furthermore, we detected a few incidents of epidemiological links between animal and human hosts. We also found that E. coli isolates from these host types were clearly mixed into local populations and that antibiotic resistance and virulence genes had been shared between strains from wild and domesticated/livestock animal hosts. These results suggest that wild hosts indeed can serve as reservoirs for E. coli pathogens and underscore the importance of large-scale population genomics studies of E. coli across multiple host species.

## RESULTS

**FIG 1:**
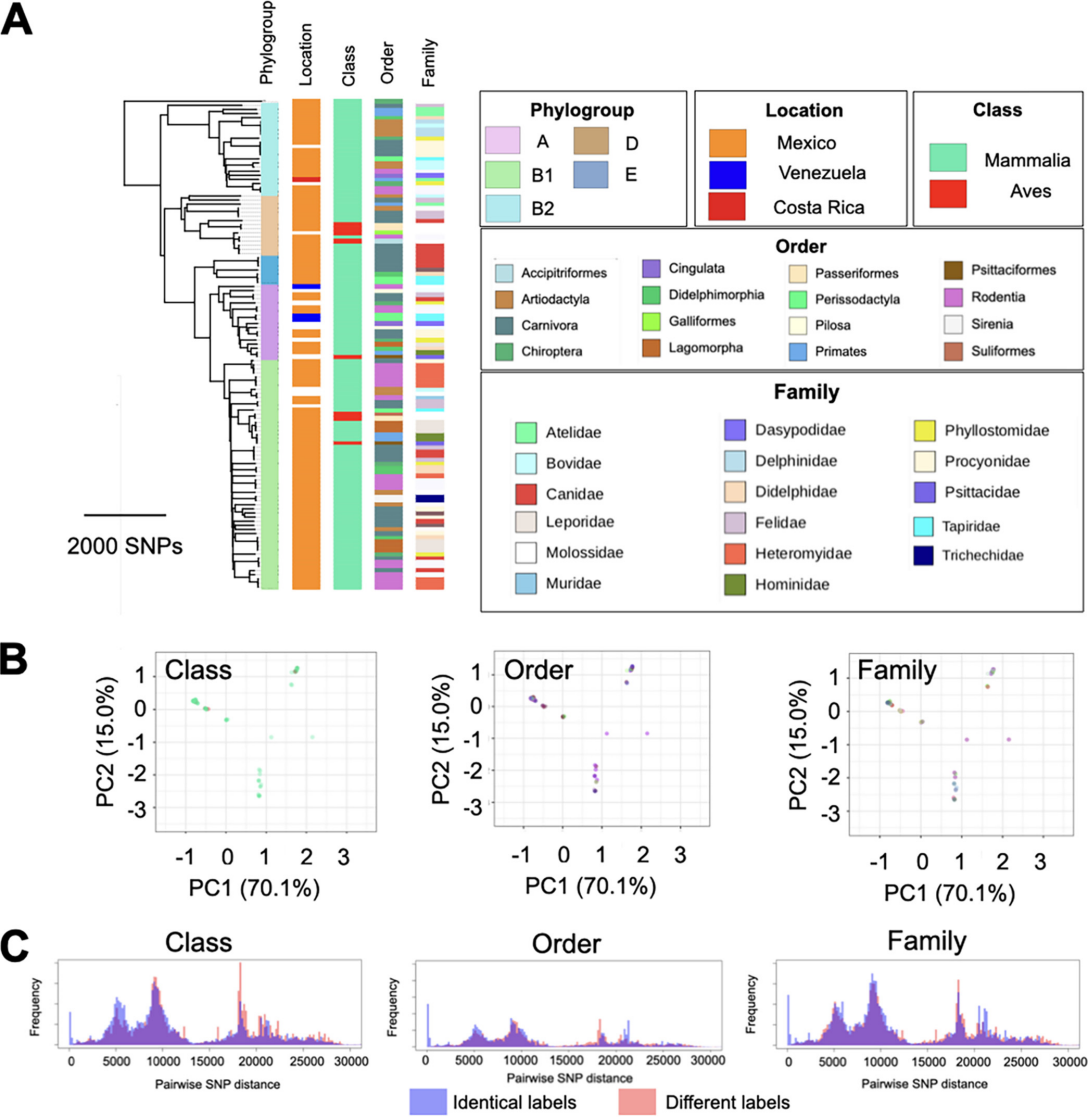
Phylogenetic distribution of host specificity and cluster analysis. (A) Phylogenetic tree of our *E. coli* strains from wild-animal hosts and its association with host taxonomy, at different taxonomic levels. Families of host species colonized by only one E. coli strain in our collection are not shown. (B) Principal-component analysis of our *E. coli* strains, with labels representing the phylogroup of the E. coli isolate and the taxonomic rank of the host species. Each color corresponds to one taxonomic rank, shown in panel A. (C) Distribution of pairwise SNP distances for E. coli strains from hosts belonging to the same (red) and different (blue) taxonomic ranks.

We sequenced 119 strains isolated from 55 wild animal host species predominantly in Mexico and found them to capture much of the known global E. coli genetic diversity. Indeed, our wild-host collection contained representatives of all of the major known phylogroups of E. coli, with group B1 (55 strains; 47% of the total) being most prevalent, followed by B2 (21 strains; 18%), A (17 strains; 14%), D (15 strains; 13%), and E (7 strains; 6%) (Fig. 1A). The high frequency of B1 strains is consistent with previous epidemiological reports on E. coli isolated from domesticated animals but stands in contrast to the high prevalence of phylogroups B2 and A among E. coli isolates from human hosts, indicating an association between the E. coli phylogenetic structure and the type of natural host colonized by E. coli (16). Analyzing the genomes of our E. coli isolates from wild hosts together with those of many previously sequenced strains with diverse origins, we found that E. coli from domesticated/livestock animals and North America were disproportionately likely to share phylogenetic origins with our wild E. coli strains (see Fig. S1A and B in the supplemental material). This suggests a regional dissemination of specific E. coli clones (sequence types) across both domesticated/livestock and wild animals in North America, which motivated us to examine the prevalence of transmissions and recent divergences at a fine resolution.

10.1128/mSphere.00738-20.1 FIG S1 Phylogeny of E. coli from wild hosts with strains from other sources. (A) Alignment-free phylogenetic tree for the E. coli strains in the context of previously sequenced genomes in the Enterobase database and the associated metadata of phylogroup and isolation sites. (B) The distributions of continent of isolation and host in the curated dataset, compared with those in the Enterobase collection. Accession number and metadata for strains are provided in Table S2. Download FIG S1, TIF file, 0.9 MB. Copyright © 2021 Murphy et al. 2021 Murphy et al. This content is distributed under the terms of the Creative Commons Attribution 4.0 International license.

**FIG 2:**
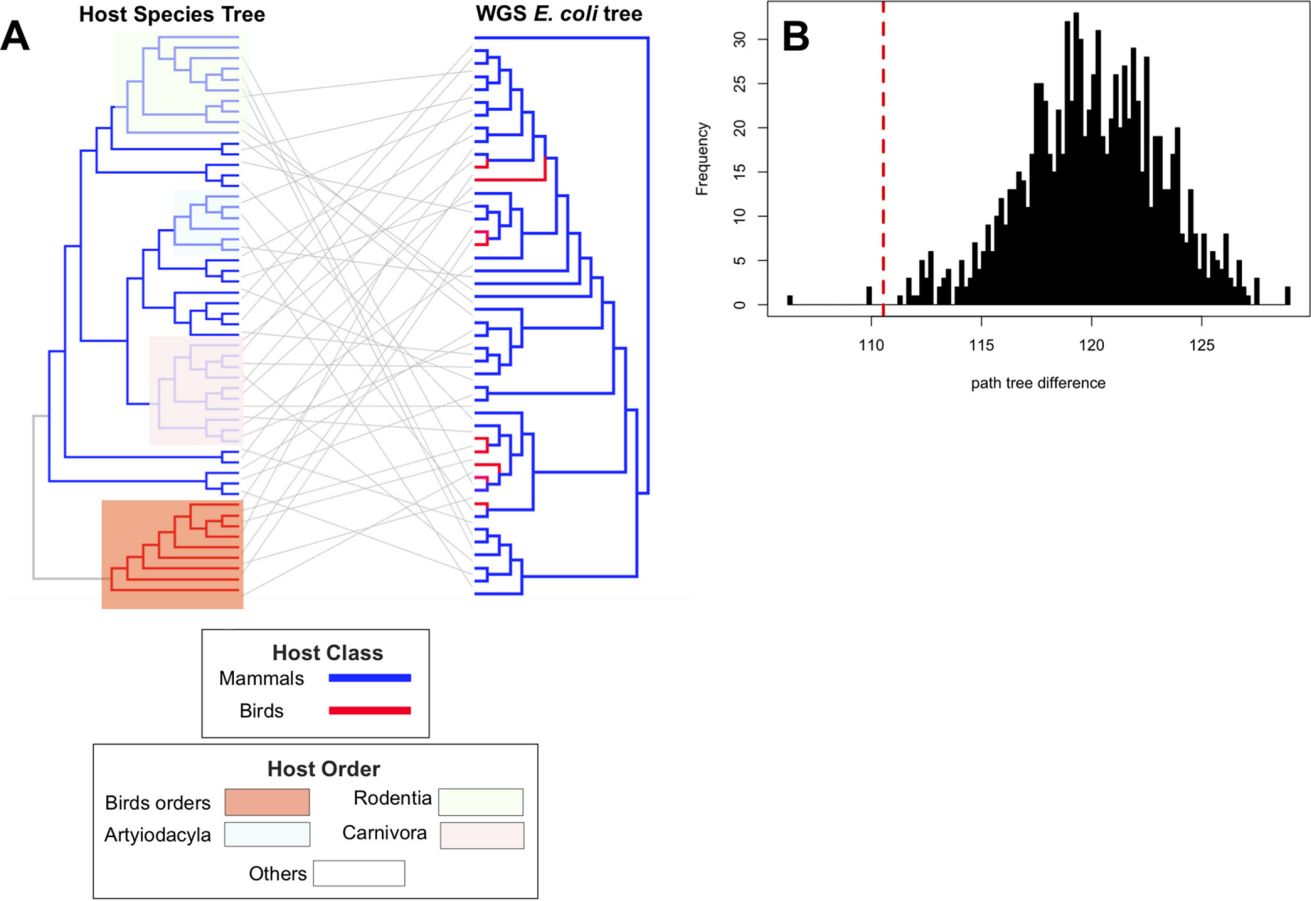
Concordance between host and E. coli phylogenetic trees. (A) Phylogenetic tree of the whole-genome sequencing of E. coli strains and the tree of life (TOL) for host strains. For host species for which more than one isolate were available in the data set, one strain was randomly drawn. Clades for bird and major mammalian orders are highlighted. (B) The frequency (*y* axis) of path tree differences relative to the *E. coli* tree (*x* axis) for 1,000 random shuffling of tree tips of the host tree in panel A (black bars), contrasted to the observed value from unpermuted data (red dashed line).

We compared the phylogenetic trees for our collection of E. coli strains from wild animals and their hosts to understand the long‐term concordance between the evolutionary histories of E. coli and their host species and the extent to which the genetic distance between the host species agrees with the regional population structure of E. coli. Both comparisons of host and E. coli distance matrices (*P = *0.0001; Mantel test) (Fig. 2A) and comparisons of distances between phylogenetic trees for E. coli strains and hosts to distances in randomized trees (*P = *0.003; 1,000 tests) (Fig. 2B) rejected completely random observations. Despite this, we found only a moderate correlation of 0.47 between the genetic distance matrices for E. coli strains and hosts (Fig. 2A), with closely related E. coli strains sometimes colonizing divergent wild hosts and closely related wild animal species sometimes hosting distantly related E. coli. The limited genetic association between E. coli isolates and their wild hosts was also evident at higher taxonomic levels, with only weak genetic clustering of E. coli according to the host class, order, and family (Fig. 1B). This was further confirmed by the extensive overlap in the distributions of single-nucleotide-polymorphism (SNP) distances for E. coli pairs colonizing host species from the same taxonomic groups and those of pairs colonizing different taxonomic groups (Fig. 1C), as 0.95, 0.95, and 0.96 of ranges of distributions overlapped for taxonomic ranks of class, order, and family, respectively. Moreover, we found the accessory genome of our E. coli colonizing wild hosts to have evolved in concert with their core genome (*P = *0.0001; Pearson’s *R* = 0.85; Mantel test on distance matrices for core genome and accessory genes) (Fig. S2). Together, these results provide evidence of regional gene flow across E. coli lineages colonizing wild-animal hosts in Mexico (see Discussion).

10.1128/mSphere.00738-20.2 FIG S2 Comparison between core genome and accessory genome trees. The core genome and accessory genome trees were reconstructed from the SNPs in the core genome alignment and from the pattern of gene presence and absence, respectively. Download FIG S2, TIF file, 0.7 MB. Copyright © 2021 Murphy et al. 2021 Murphy et al. This content is distributed under the terms of the Creative Commons Attribution 4.0 International license.

Host adaptation is a consequence of diversifying selection across lineages, but it may be influenced by random effects due to, e.g., population structure (17). To examine the extent to which selection has shaped genetic variation in our E. coli collection from wild-animal hosts, we compared the rates of nonsynonymous and synonymous single-nucleotide evolution (*K_a_*/*K_s_*) since their last shared common ancestor. Of 3,529 genes in the core genome, 242 had a *K_a_*/*K_s_* value above 1 in at least one strain, with an average of 11.7 genes, i.e., 0.3% of total genes, per strain falling in this category (Fig. S3A and B; Table S3). The number of genes under strong positive selection did not show any link with the type of wild-animal host from which the strains had been isolated (Fig. S3B). The strongly selected genes encoded proteins involved in a broad range of functions, with genes encoding energy production, carbohydrate and ion metabolism and transport, and signal transduction proteins being slightly overrepresented (Fig. S3B). This pattern agrees with a complex nature of the E. coli adaption to colonizing the guts of different wild-animal hosts and the degree of genome-wide selection having been little influenced by the type of host species colonized.

10.1128/mSphere.00738-20.3 FIG S3 Positive selection analysis. (A) The distribution of functional classifications for 256 genes that were under positive selection, compared with the baseline distribution in the pan genome. We excluded genes without assigned COG group. The letters S, G, P, C, H, and T represent unknown functions, carbohydrate metabolism and transport, inorganic ion transport and metabolism, energy production and conversion, coenzyme metabolism, and signal transduction, respectively. The full interpretation for other classes is found in reference 61. The asterisk sign shows 0.05 significance from Student’s t-test to assess the significance of the difference between groups. (B) Distribution of genes under positive selection across taxonomic host orders. Download FIG S3, TIF file, 0.5 MB. Copyright © 2021 Murphy et al. 2021 Murphy et al. This content is distributed under the terms of the Creative Commons Attribution 4.0 International license.

**FIG 3:**
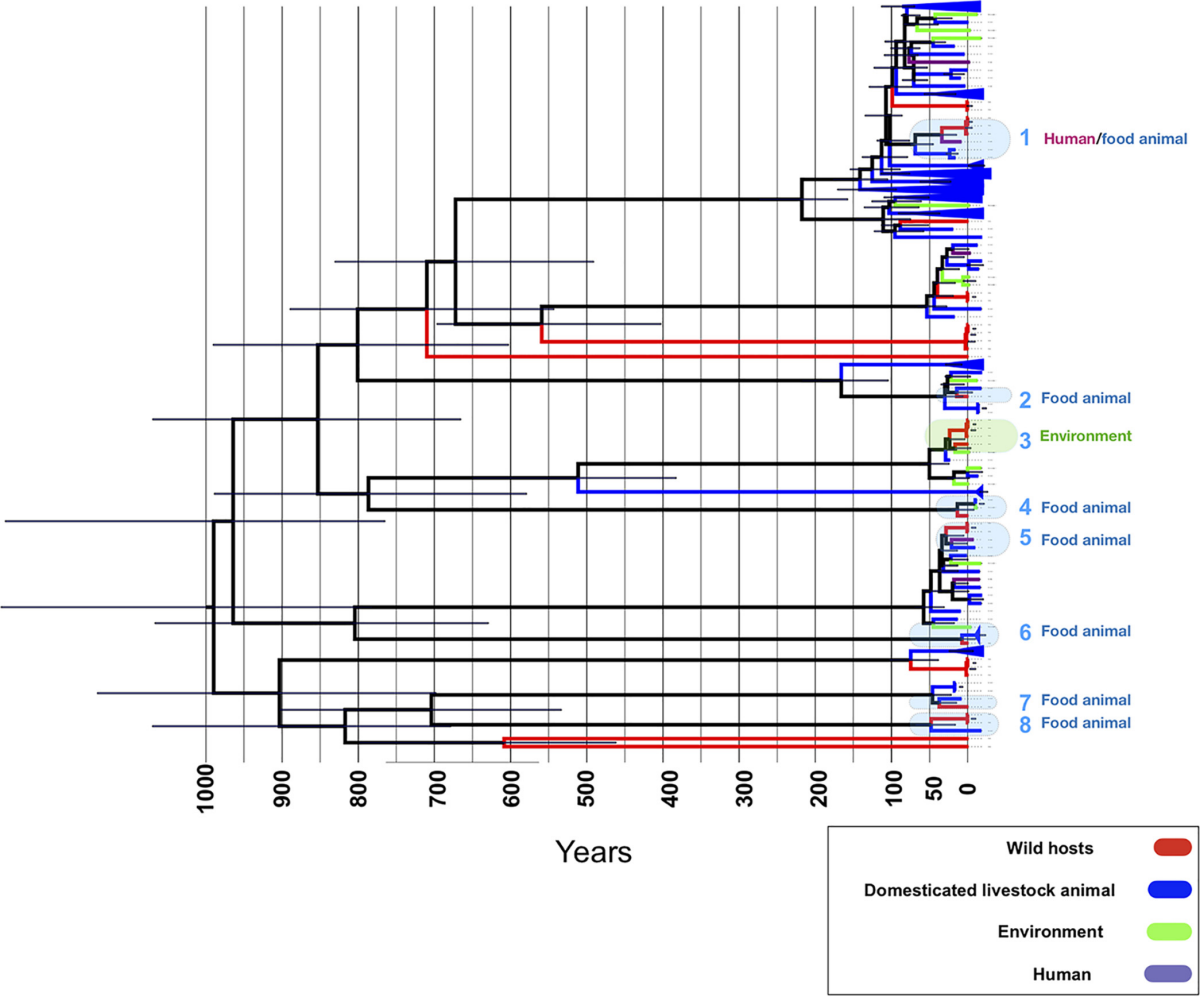
Recent mixing of wild and nonwild host lineages. Bayesian tree for strains in a clade belonging to phylogroup B1. The shaded boxes show putative host jump events between wild hosts and other sites, i.e., domesticated animals, environment, and humans, over the past 100 years. The error bar shows the 95% confidence interval from the Bayesian tree.

We next probed the genome evolution and epidemiology of our E. coli isolates colonizing wild animals in relation to those of the external collection of E. coli isolates coming from other hosts. Our analysis did not identify E. coli isolates with zero, or close to zero, SNP distance within wild hosts or food animal sources, which would suggest direct, recent transmissions. However, the analysis of SNP clusters revealed two links between wild hosts and clinical strains. One link weakly (38 SNPs) connected a sample (SAMEA4607586) from the avian species Aratinga canicularis to two clinical strains from Mexico (SNP cluster ID PDS000073768.1), and one moderately strongly (17 SNPs) connected an E. coli isolate from the avian species of Gallus gallus and 7 clinical strains (SNP cluster PDS000066827.4). One and nine of the clinical strains were from France and the United States, respectively. Interestingly, of 22 external strains isolated from wild hosts in Mexico, 17 formed parts of SNP clusters with our strains, showing extensive circulation of E. coli across wild hosts in Mexico (Table S1). The phylogenetic analysis revealed genetic similarity between our E. coli isolates colonizing wild animals and E. coli isolates colonizing domesticated animals in the B1 phylogroup, where one-third of our E. coli isolates from wild animals clustered with external lineages isolated from domesticated/livestock animals (*n *=* *96), food (*n *=* *12), and environmental sources (*n *=* *13) (see Materials and Methods). We reconstructed the Bayesian tree of these 158 strains and found their last common ancestor to have lived about 1,000 years ago, with a substantial expansion of the clade over the past 100 years (Fig. 3). We identified eight incidents of strains jumping between wild animals and other sources in this clade, all during the last 100 years and all but one during the last 50 years (Fig. 3). One incident involved E. coli jumping between wild hosts residing in city regions and domesticated/livestock animals. These E. coli host switches between wild animals and other sources may reflect anthropogenic intervention in the habitats of their wild hosts, and the rapid urban and agricultural growth and environmental degradation in Mexico over the past decades (18).

10.1128/mSphere.00738-20.6 TABLE S1 Samples specification, serotypes, associated pathovars, and virulence and AMR genes for our strains and accession numbers for external strains from wild hosts in Mexico, retrieved from NCBI. Download Table S1, CSV file, 0.1 MB. Copyright © 2021 Murphy et al. 2021 Murphy et al. This content is distributed under the terms of the Creative Commons Attribution 4.0 International license.

**FIG 4:**
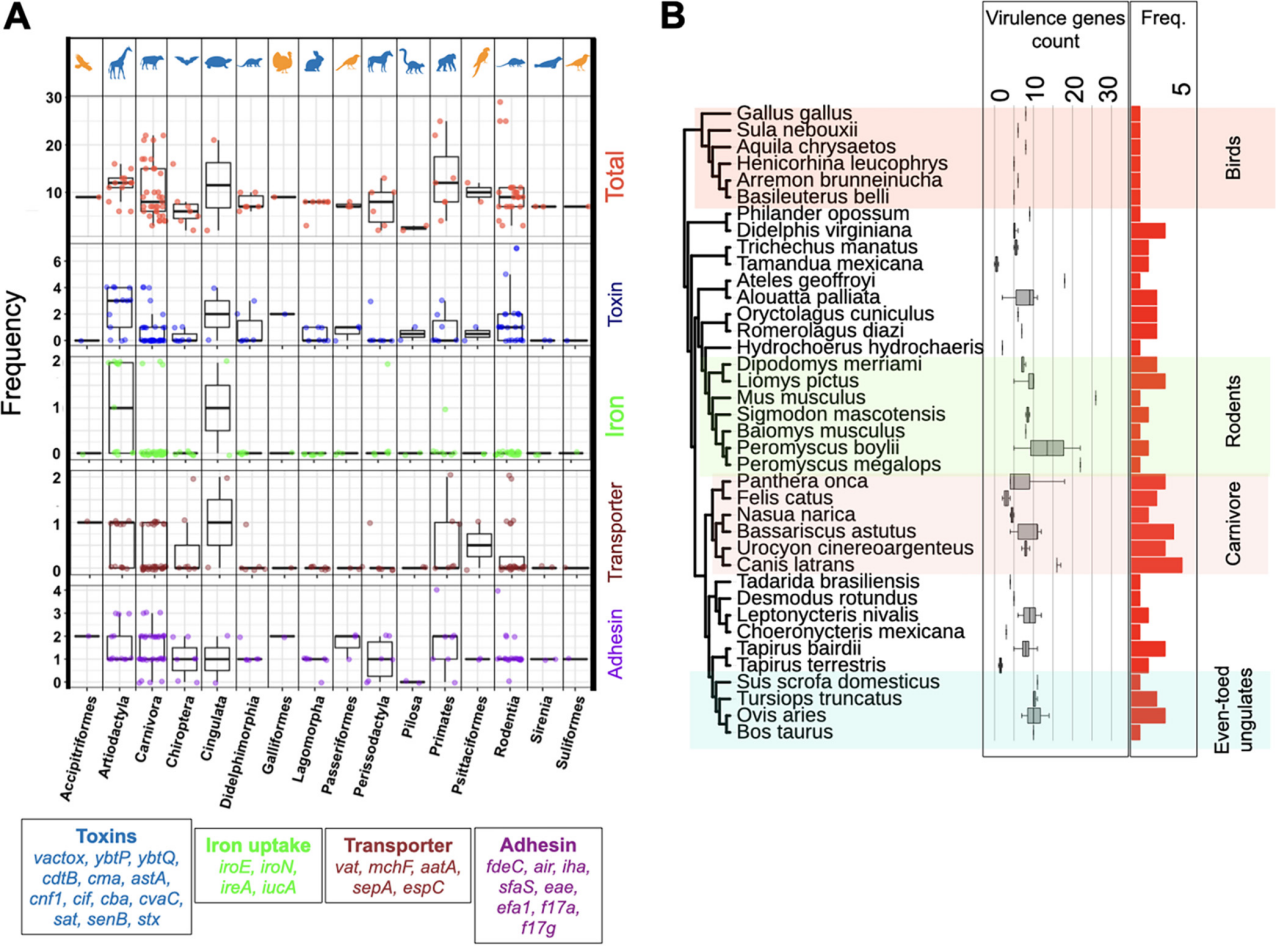
Distribution of virulence factor genes. (A) Frequency of virulence factors genes across functional groups and taxonomic orders. (B) Phylogenetic distribution of E. coli virulence genes across wild animal host species. The tree shows the tree of life for hosts, where major orders are shown in shaded boxes. Bar plots show the frequency of genes. Horizontal box plots represent the distribution of virulence genes for strains recovered from each host across host orders.

The incidents of E. coli jumping between wild and domesticated animals led us to examine whether E. coli isolates colonizing the former harbor any known human- or food-animal-linked virulence factors. We identified a range of virulence factor genes, including four types of toxin genes, two adhesin genes, two iron chelators, and three transporters. These were present in E. coli isolates colonizing different wild animals (Fig. 4A). The frequency of virulence factors was on average higher for strains recovered from Primate (11.5 genes per isolate), Rodentia (9.5 genes per isolate) and Carnivora (12.5 genes per isolate) host species (Fig. 4A and B). Some host species not closely related to humans, such as avian species, were colonized by strains carrying a high number of virulence factors (Fig. 4A and B), suggesting that the pattern is not simply a reflection of the higher frequency of human- and livestock-associated genes in the database.

Because both the physiology and ecology of the host species can affect the virulence factors encoded in the genomes of infectious bacteria, we examined the relationship between the number of virulence genes in E. coli isolates colonizing wild animals and the 45 such features in the panTHERIA database. A previous study on four virulence genes revealed that the body mass of the host species can be positively linked with the number of virulence factors present in the gut microbiome, and this was attributed to the gut complexity (19). However, our analysis of many more virulence genes showed no such correlation, considering either adult, neonate, or weaning body mass (Fig. S4A). Only the terrestriality, i.e., adaptation to living on land (*P = *0.03; Spearman's ρ = −0.18), habitat breadth (*P = *0.05; Spearman's ρ = −0.24), diet breadth (*P = *0.02; Spearman's ρ = −0.26), and social group size (*P = *0.02; Spearman's ρ = 0.27) of hosts correlated significantly with E. coli virulence gene counts. More diverse habitats and diets of the hosts were associated with fewer virulence genes, and the formation of larger social groups was associated with more virulence genes in E. coli isolates colonizing these hosts (Fig. S4A and B). Larger social groups, as observed in Carnivora, Artiodactyla, and Primates (Fig. S4C), are known to increase the social transmissions of infectious agents, such as E. coli, in animal societies, which may facilitate the dispersion of virulence genes among these infectious agents (20). Although a larger sample set is needed to examine the impact of potential confounding factors, the findings further support the idea that a complex network of host- and environment-related factors shapes the genomic characteristics of commensal E. coli strains.

10.1128/mSphere.00738-20.4 FIG S4 Correlation between virulence factor counts and physiological and ecological attributes of wild hosts. (A) Correlation coefficient between the number of virulence genes and ecological features in the panTHERIA dataset. The size and color of the circles correspond to the absolute value and direction of Spearman’s rank correlation coefficient, respectively. Entries with insignificant correlation correlations, i.e., *P* values of <0.05, are shown as blanks. The red box shows the ecological features that are significantly correlated with virulence factor counts. (B) Correlation between social group size and total number of virulence genes for the wild-host E. coli sample set. The blue line is the fitted linear regression model. The grey area corresponds to the 95% confidence interval. The coefficient is 0.22419, with a standard deviation of 0.09071. (C) Social group size values across host orders. Download FIG S4, TIF file, 0.6 MB. Copyright © 2021 Murphy et al. 2021 Murphy et al. This content is distributed under the terms of the Creative Commons Attribution 4.0 International license.

**FIG 5:**
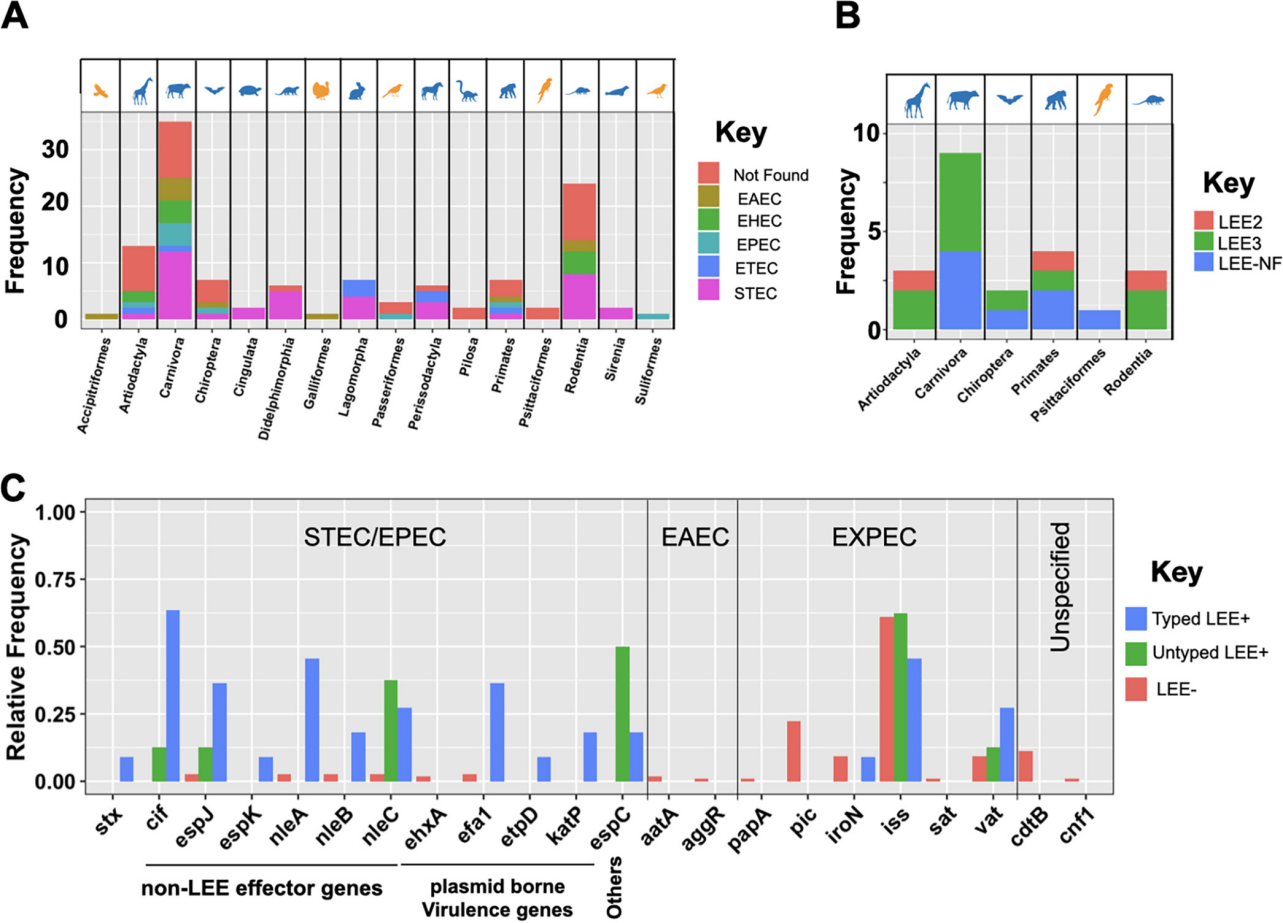
Sharing of serotypes and distribution of LEE genes and effectors genes across hosts. (A) Distribution of serotypes shared between E. coli isolates colonizing wild hosts and known pathovars across taxonomic orders of hosts. (B) Distribution of typed and nontyped LEE families across taxonomic orders of hosts. (C) Distribution of virulence genes and LEE effector genes in typed LEE-positive, untyped LEE-positive, and LEE-negative strains.

Certain E. coli serotypes, which reflect O, H, and K antigen variation, are recognized to cause virulence in human- and livestock-associated infection. We found 53 and 14 serotypes to be shared between our E. coli strains colonizing wild animal hosts and those in domestic animal and human infections, respectively (Table S1). In total, we identified 71 distinct serogroups and 14 strains among E. coli isolates colonizing wild animals that were not typeable according to known serotype patterns, further underscoring their broad diversity. The serogroups of 74 strains overlapped with those of known pathovars, including non-O157 Shiga toxin-producing E. coli (STEC) (*n *=* *40), enterotoxigenic E. coli (ETEC) strains (*n *=* *12), enteropathogenic (EPEC) strains (*n *=* *11), and enteroaggregative E. coli (EAEC) strains (*n *=* *11), across hosts (Fig. 5A; Table S1). The pathovars are recognized to have nonhuman sources and are known to be acquired by humans via direct contact with either animals or their feces, in petting zoos and on farms (for STEC), or through the consumption of contaminated water and food (for EAEC and ETEC), as previously reported in Mexico (21, 22). ETEC is also an important cause of diarrhea in domestic animals, notably in calves and piglets (23). Two strains from our wild host collection shared serotypes with human-pathogenic strains and contained the genetic virulence hallmarks of their associated pathovars. One strain belonged to O111:H8, a clinically relevant enterohemorrhagic E. coli (EHEC) serotype, and contained both the enterocyte effacement (LEE) pathogenicity island (PAI) and the toxin *stx*_2_ gene. This strain was recovered from a wild sheep close to a city. The other strain belonged to O78:H34 and was isolated from a parakeet carrying EAEC virulence genes, including the gene for the plasmid-encoded, heat-stable enterotoxin toxin (EAST-1) and *aatA* and *aggR*, encoding a transporter of a virulence protein and a virulence regulator, respectively (Fig. 5). The serogroup was recently isolated from free pigeons in Brazil, showing the circulation of the pathovar among birds (24). None of the 74 strains whose serogroups were associated with STEC and ETEC pathovars were found to carry a toxin gene. Since our strains were recovered from feces, the virulence ability of their shared serotypes outside the gut in wild hosts is unclear. However, our findings are in line with the idea that virulent pathovars in food animals have emerged as a result of the acquisition of virulence factor genes by isolates belonging to serotypes of wild-host origin.

We found the pathogenicity island LEE, which is a hallmark of STEC and EPEC pathovars, in 21 of the E. coli lineages from wild-animal hosts, and these hosts belonged to six different taxonomic orders (Fig. 5B; Table S1). The LEE encodes factors required for the colonization of the human intestine (25). However, the absence of the plasmid carrying E. coli adherence factors (pEAF) led us to classify these isolates as atypical EPEC (aEPEC), an E. coli class that is widely spread across food animals and humans (26). Our LEE-positive E. coli strains also harbored other virulence factors that are typical of EAEC and extraintestinal pathogenic *E. coli* (ExPEC) pathovars and that affect pathogenicity (Fig. 5C). This included genes normally located on STEC virulence plasmids, such as pO157, pO26, *espP*, and *nle*, all of which were significantly more frequent among our LEE-positive strains than among our LEE-negative strains (*P* < 0.001 for two-sided Bonferroni-corrected Fisher's exact test) (Fig. 5C). We found that 2 and 11 strains, all in the B1, E, and D phylogroups, carried the LEE2 and LEE3 variants, respectively, while 8 strains, mainly in the B2 phylogroup, carried a nontypeable LEE. All three locus types were broadly distributed among host taxonomic families, in agreement with their benefitting E. coli colonization of animal guts in a general sense, as previously proposed for bovine hosts (13). Our findings also agree with the virulence ability of aEPEC strains spanning a broad host range and with the idea that virulence in STEC and EPEC strains has evolved by commensal strains acquiring virulence factors sequentially (26). We also identified a set of ExPEC-associated genes, encoding toxins (*pic*, *sat*, and *vat*), iron uptake (*iroN* and *iha*), and serum resistance (*iss*) proteins (Fig. 5C). Whether the presence of these genes is sufficient for a strain to cause sepsis or bacteremia is unclear, since the transition between asymptomatic colonization of the guts to the spreading of the bacteria into the bloodstream strains is poorly understood for ExPEC strains (27).

We tested the sensitivity of our collection of E. coli isolates from wild animal hosts to antibiotics in common use against human E. coli infections and found them to be mostly sensitive, except that 65% of the strains were resistant to ampicillin (Table S1). Their general susceptibility to antibiotics agrees with the lack of historical exposure of E. coli colonizing wild animals to therapeutic levels of antimicrobials. However, despite their general sensitivity to antibiotics, we found a range of AMR genes against beta-lactamase, aminoglycosides, sulfonates, and ciprofloxacin in the genomes of different E. coli lineages colonizing different host species (Fig. S5). This discordance between AMR phenotypes and genotypes points to regulation mechanisms or other epistatic effects that reduce the phenotypic penetrance of these resistance genes. The genomic context of these AMR genes turned out to be diverse, with genetic linkage to a range of phage genes and insertion sequence (IS) elements, including to IS91 and IS10. For AMR genes located on sufficiently long contigs, we explored the genomic context and found similarity with broad-host-range Col plasmid (*n *=* *21) and chromosomal (*n *=* *3) regions. The genomic contexts varied across host species; for example, while one strain from a member of the Pilosa, a placental mammal, harbored a distinct AMR gene cassette consisting of *tet*, *str*, and *sul* genes, we found the AMR genes of four other strains, from different mammalian species, to be sporadically distributed across the genomes. Besides plasmid-borne resistance determinants, we identified a set of ciprofloxacin resistance mutations in the *parE*, *parC*, and *gyrA* genes which emerged independently across lineages (Fig. S5). The strains had been recovered from Carnivora, Rodentia, and Passeriformes species. Four of the isolates belonged to the clinically relevant O17/77:H18 serotype, which forms a highly relevant pathogenic group in phylogroup D that was a clinical threat in the 1990s, predominantly in North America (28). Ciprofloxacin was introduced into clinical settings in the 1980s (29), prior to the sampling time period of our collection. The presence of ciprofloxacin resistance determinants in wild hosts, therefore, suggests that either rapidly emerging resistance was transmitted from wild hosts into human settings prior to the sampling time period or resistance preexisted in wild-host reservoirs.

10.1128/mSphere.00738-20.5 FIG S5 Phylogenetic distribution of antimicrobial resistance genes.: The tree was built from SNPs in the core genome, using the neighbor-joining method. For ciprofloxacin resistance, chromosomal mutations are shown. Download FIG S5, TIF file, 0.5 MB. Copyright © 2021 Murphy et al. 2021 Murphy et al. This content is distributed under the terms of the Creative Commons Attribution 4.0 International license.

## DISCUSSION

We examined a collection of E. coli strains in wild hosts in Mexico to understand the regional genomic epidemiology of these strains. We integrated available data on the host species with the E. coli whole-genome sequencing data to understand the host-associated population structure in the collection. Despite the limited size of the collection and its regional nature, we found it to be genetically diverse, containing representatives from all major phylogroups of E. coli. We also found some of our strains to belong to local E. coli populations also colonizing lineages of domesticated/companion animals in the region. Moreover, some of our wild-animal strains harbored virulence and AMR genes that they shared with lineages identified as pathogenic to human and livestock animals.

The absence of strong evidence for transmission of E. coli from wild animals, predominantly in Mexico, to human hosts suggests that wild hosts are not immediate infection sources in human outbreak networks in the region. However, as our sampling of E. coli isolates colonizing wild animals in the region is far from exhaustive, we cannot exclude the possibility that such transmissions have occurred. The problem of nonexhaustive sampling is prevalent in genomic epidemiological studies, most of which have reported a clear genetic distinction between E. coli isolates found in humans, food animals, and other sources and few incidents of E. coli transmission between them (30–32). The problem is an issue not only of sample size but also of the breadth of sources from which they are obtained, and expanding both such that strong conclusions can be drawn from these negative results will remain a challenge.

We note that since our strains were recovered from feces, we are unable to ascertain whether the presence of pathovar-associated genes in human and livestock strains is sufficient to cause virulence, when introduced into the bloodstream in wild hosts. However, our findings are consistent with E. coli isolates that colonize wild-animal hosts serving as a source/sink for known pathogenic strains, serotypes, and genes. Nonhuman origins for human EPEC strains were reported in a recent large-scale genomic study, but the study examined only livestock sources (13). Our results complement these findings and suggest that evolution of virulent strains in some cases can be traced back to wild-animal sources, highlighting the role of E. coli host diversity in facilitating human infections. Besides virulent strains, our results demonstrate that wild animals serve as reservoirs for antibiotic resistance and virulence genes and that these can be transmitted to strains acting as human pathogens. This is in line with a recent study that also demonstrated a major role of horizontal gene transfer (HGT), through mobile genetic elements, in the recent spread of beneficial E. coli genes across niches in Australia (8). A more dominant role of HGT, compared with mutations arising *de novo* in the core E. coli genomes, was also shown in an *in vivo* study on the colonization of the mouse gut by commensal E. coli strains (33).

Besides the limitations in scope imposed by sample size, we did not examine the intrahost diversity of E. coli strains. Genetically distinct strains reside within the gut, and the genetic composition of E. coli genomes varies systematically across the different regions of the gut. One or two resident E. coli clones most often dominate the microbial community of the gut of any one individual (34), and the strains recovered from each species in our study are likely to correspond to one such clone. However, different clones may dominate in different individuals of the same species, and a broader sampling of each host species is therefore required to better understand the E. coli-host species interaction. Multisite sampling of individuals would also allow us to examine whether virulence genes present in the dominant clone confer any fitness advantage over other clones and whether these advantages persist across the species or even across larger taxonomic distances. Our study also neglected the degree of expression of antibiotic and virulence genes (35), which helps determine the extent to which they exert their function and which could shed light on the discordance between the presence of such genes in the genome and the absence of an evident functional activity, in terms of antibiotic resistance.

Studies on E. coli genomics have largely focused on pathogenic clinical strains under therapeutic conditions. However, to understand the evolutionary trajectories from commensalism to pathogenicity, we must also decipher the genetics of commensalism. Ours is one of a growing number of studies that focus on E. coli’s nonhuman natural habitats and that seek to describe the distribution of genes and properties in the global population of E. coli strains. The insights from these studies not only facilitate the diagnosis and tracking of infectious E. coli strains at an epidemiological level but also may help to pinpoint genetic biomarkers for pathogenicity, which are potential targets for the development of therapeutic agents.

## MATERIALS AND METHODS

### Strain acquisition, sequencing, and genome assembly.

We acquired a systematic collection of commensal E. coli isolates from a previous study (5). The collection comprised 119 fecal strains from hosts belonging to 55 animal species, 31 families, and 16 orders. Of these, 110 and 9 strains were from mammals and birds, respectively. Also, 110 strains were recovered from Mexico and the rest were isolated in Venezuela and Costa Rica during the 1990s. The antimicrobial susceptibility testing was conducted on the whole collection for 8 antimicrobials clinically approved for treating E. coli infections, including beta-lactams (ampicillin, cefotaxime, ceftazidime, cefuroxime, and cephalothin), aminoglycosides (gentamicin and tobramycin), ciprofloxacin, and trimethoprim, as described in reference 36. The full description of the strains with metadata is available in Table S1.

DNA was extracted with the QIAxtractor (Qiagen) kit according to the manufacturer’s instructions. We prepared Illumina sequencing libraries with a 450-bp insert size and performed sequencing on an Illumina HiSeq2000 sequencing machine with paired-end read lengths of 100 bp. Ninety-six samples were multiplexed to yield an average depth of coverage of ∼85-fold. Reads were then assembled and improved with an automated pipeline, based on Velvet with default parameters. Assemblies were annotated with an improvement assembly and Prokka-based annotation pipeline, respectively (37–39). Details on assembly statistics, access codes for annotated assemblies, and gene annotations are available in Table S1. Roary, with the sequence identity value of 95% for orthologous groups, was used to create a pan-genome from annotated contigs (40). The Roary output file is available on the GitHub directory for the project (www.github.com/dmoradigaravand/WildHostEcoliMexico). Roary identified 24,060 genes, composed of 2,855 genes in the core genome (present in at least 99% of strains), 357 genes in the soft core genome (present in 95% to 99% of strains), 2,141 genes in the shell genome (present in 15% to 95% of strains), and 18,707 genes in the cloud genome (present in up to 15% of strains). Multilocus sequence typing was performed on assemblies using a publicly accessible typing tool and database (www.github.com/sanger-pathogens/mlst_check) with default parameter values to identify sequence type (ST) clones. Multilocus sequence typing (MLST) results are provided in Table S1. We identified phylogroups using ClermonTyping (41).

We contextualized our collection with E. coli strains from the environment, livestock/domesticated animals, and humans in the publicly available Enterobase data set (https://enterobase.warwick.ac.uk/). Since we were primarily interested in recent evolution and transmissions between E. coli in wild hosts and other hosts, we retrieved genomic data and metadata for all strains with an identical ST with at least one strain in our collection on 26 April 2020. We included only strains for which prior consent was obtained from the strain’s owners. In total, genomic data for 1,868 strains were retrieved. The accession numbers and associated metadata are provided in Table S2. We then classified strains based on their source of isolation, i.e., environment, livestock/domesticated animals, and human associated. We used the above-mentioned pipeline to assemble the paired-end short reads and annotate the assemblies for external samples.

10.1128/mSphere.00738-20.7 TABLE S2 Sample specification and associated metadata for external isolates from Enterobase, shown in Fig. S1. Download Table S2, CSV file, 0.1 MB. Copyright © 2021 Murphy et al. 2021 Murphy et al. This content is distributed under the terms of the Creative Commons Attribution 4.0 International license.

10.1128/mSphere.00738-20.8 TABLE S3 *K_a_*/*K_s_* values and functional annotations for genes present in >70% of isolates. Download Table S3, CSV file, 0.2 MB. Copyright © 2021 Murphy et al. 2021 Murphy et al. This content is distributed under the terms of the Creative Commons Attribution 4.0 International license.

We also examined the genetic relatedness between our strains and 138,507 E. coli strains, available on the NCBI Pathogen Detection database (www.ncbi.nlm.nih.gov/pathogens) on 4 December 2020. We determined whether our strains fell in any SNP cluster, i.e., isolates that differ by <50 SNPs, with external strains from wild-animal, food animal, and human hosts. Assuming a substitution rate of two SNPs/year (42) for E. coli genomes, the SNP distance of 50 corresponds to 25 years. The accession numbers for these clusters, which are indicative of recent divergences, are provided in Table S1. In addition to our strains, we also examined a total of 22 external strains from wild hosts of Mexican origin in the NCBI database to detect whether they are closely related to any other strain from human or food animal hosts. The accession numbers for these strains and their origins are provided in Table S1.

### Mapping, variant calling, and phylogenetic analysis.

We mapped short-read sequences to the E. coli K-12 sequence (Biosample ID SAMN02604091), with SMALT v 0.7.4 (https://www.sanger.ac.uk/tool/smalt-0/), with a minimum score of 30 for mapping. SAMtools and BCFtools were then employed to annotate SNPs (43). SNPs at sites in which SNPs were present in less than 75% of reads were excluded. We extracted SNPs from the core-genome alignment produced by Roary and mapped them to the E. coli K-12 reference genome using the script available at https://github.com/sanger-pathogens/snp-sites. The SNP alignment file for the core genome is provided in the GitHub directory (www.github.com/dmoradigaravand/WildHostEcoliMexico).

Because E. coli genomes are too divergent to map to a single genome, we adopted an alignment-free approach to analyze the phylogenetic tree for the collection composed of wild-type and external strains. To this end, we first enumerated *k*-mers with a size of 50 from assemblies with the frequency-based substring mining (fsm-lite) package (www.github.com/nvalimak/fsm-lite). We subsequently counted the number of identical *k*-mers for pairs of isolates to produce a similarity matrix, which was then converted into a distance matrix. The distance matrix was used as input for the ape (44) and phangorn (45) packages to produce a neighbor-joining phylogenetic tree. The tree was visualized with iTOL (46) and Figtree (http://tree.bio.ed.ac.uk/software/figtree/).

### Virulence factors, antimicrobial resistance gene identification, and *in silico* serotyping and LEE typing.

Virulence factors and antimicrobial resistance genes were identified with the VirulenceFinder and ResFinder (both chromosomal and plasmid-borne genes) online servers (https://cge.cbs.dtu.dk/services/) using the Virulence Factor Data Base (VFDB) (47) and ResFinder database (48), respectively. We employed a loose similarity and a minimum length cutoff of 60% to ensure that divergent genes were detected. We merged the set of identified genes with those reported on the NCBI Pathogen Detection database, i.e., AMRFinderPlus, for our strains (49). We used the virulence gene panels defined in references 13 and 27 for E. coli pathovars to determine the pathogenicity of strains from human and food animals. We provide the list of identified resistance and virulence factors in Table S1.

The genomic context of the AMR genes was explored in two ways. First, we searched the nucleotide database to find similar annotated genomic regions with the contig that contains the resistance gene with BLASTN. Second, to further examine whether genes are located on the plasmid or the chromosome, we also utilized PlasmidSPAdes (version 3.9.0) (50) to first reconstruct plasmid assemblies and then screened the contigs for the AMR gene with BLAST, as part of the assembly graph viewer Bandage (51). We identified LEE and serotypes with the typing method in the srst2 package, using a similarity threshold of 60%. We then confirmed the presence of virulence factor genes by running BLASTN against assemblies. For the O antigens produced by the Wzy-dependent pathway, variations in the unique genes *wzx* (encoding an O-antigen flippase) and *wzy* (encoding an O-antigen polymerase) were examined (21). For the ABC transporter-dependent pathway, variations in *wzm* (encoding an O-antigen ABC transporter permease gene) and *wzt* (encoding an ABC transporter ATP-binding gene), involved in O-antigen synthesis, were studied. All the databases for typing are available in the srst2 package, and results of typing are provided in Table S1. To link serotypes with pathovars, we conducted a search in available online literature sources on 26 April 2020 to find whether the serotype has been reported in association with a pathovar. The links to sources are provided in Table S1.

### Association with ecological and taxonomical attributes of host species.

We obtained the tree of life for the wild host species with the R package rotl (22) and visualized the concordance between the host tree and the core genome tree of colonizing E. coli strains with Dendroscope (52). We used the treedist function in the ape package to compute the distance matrix from the phylogenetic tree. For E. coli strains, the distance matrix was obtained from pairwise Hamming distances between core genome sequences. We then used a Mantel test with 1,000 permutations as part of ade4 package (53) to assess the correlation between the distance matrices for E. coli genomes and that for host species. To compute the difference between the phylogenetic trees of E. coli strains and hosts, we used the treedist function, as part of the phangorn package in R. By doing so, we computed the square root of the sum of squares of differences in path length between each pair of tips in two trees (54). The path is defined as the number of edges within the tree that must be traversed to navigate from one tip to the other.

We dissected the relationship between virulence ability, measured as the total number of virulence genes, and ecological and physiological attributes of each host species in the panTHERIA database (55). The database includes a comprehensive species-level data set of life history and ecological and geographical traits of all known extant mammals. Spearman’s rank correlation coefficient values were computed to assess the significance of the correlation between virulence gene count and attributes.

### Positive selection analysis.

We analyzed positive selection by reconstructing the ancestral sequence for each gene in the core genome, identified by Roary, with FastML using the general time-reversible (GTR) model as the evolutionary model for nucleotide substitution (56). Subsequently, the seqinR 1.0–2 package (57) was employed to compute the *K_a_* and *K_s_* values for each strain, in comparison to the ancestral sequence. We left out the strains with no synonymous changes, i.e., a *K_s_* of 0. For functional enrichment analysis, COG (clusters of orthologous groups) categories of genes were extracted from the annotation by Prokka and assigned to functional classes. We used COG categories (functional groups) for Escherichia coli K-12 substrain MG1655 on https://www.ncbi.nlm.nih.gov/research/cog/. We repeated the analysis on genes that were present in 3,529 strains, which corresponds to >70% of strains. The results of positive selection analysis for these genes are detailed in Table S3.

### Bayesian analysis.

We constructed a Bayesian tree using BEAST (58) to date the recent mixing between E. coli from wild hosts and other strains in a clone in the B1 phylogroup. The clone was identified with the clustering tool in the adegenet package (59). To this end, we used the pairwise SNP distance matrix, reconstructed from multiple alignment of genomes mapped to the reference genome. We then used the gengraph function as part of the adegenet package to compute the clusters in the population. The function is based on hierarchical clustering of the pairwise SNP distance measures and involves a hyperparameter for the number of clusters. To tune the hyperparameter, we screened the SNP cutoff value for identifying clusters in the wild host and global collection and used the clustering that remained unchanged for the highest number of SNP cutoff values. As a result, the most robust clones were identified. In total, we found 90 clusters, including 14 clusters consisting of a total of 158 strains. These 14 clusters belonged to the phylogroup B1 and contained a high number, i.e., 30/119, of E. coli strains from wild hosts. We then extracted the genomes of these strains from the multiple alignment.

The multiple alignment encompassed 2,176 variant sites and included 128 strains from the global collection. We ran Gubbins (60) with 5 iterations to remove hypervariable sites from the genome alignment and produced a neighbor-joining phylogenetic tree. To assess the strength of the temporal signal, we plotted the root-to-tip distance versus year of isolation and performed 10,000 bootstraps with randomized years to attain a distribution for *R*^2^ values. Subsequently, we compared the *R*^2^ value for the data distribution with the simulated distribution. The temporal signal for the data set was stronger than 95% of signals for bootstrapped samples. We provided the SNP alignment file for the strains the clade in the GitHub directory (www.github.com/dmoradigaravand/WildHostEcoliMexico).

The multiple alignment was then used as input for BEAST. We examined a range of prior models, including a strict molecular clock and a log-normal model of a relaxed molecular clock with constant population size. Markov chain Monte Carlo (MCMC) simulations were performed three times for 50 million generations with sampling every 10 generations. A cutoff 100 was chosen for the effective sample size (ESS) of key parameters, i.e., the substitution rate, the tree root height, and the population size, for the convergence of simulations. We used TreeAnnotator v1.10.4 to aggregate trees after removing 0.2 of the tree as the burn-in phase. The 95% highest posterior interval (HPI) was used to report the certainty on ages of ancestral nodes.

### Data availability.

Short-read data were submitted to the European Nucleotide Archive under the BioProject accession number PRJEB23294.
