# Supplementary figures and images for: Genomic Epidemiology and Evolution of Escherichia coli in Wild Animals in Mexico
Source: mSphere. 2021 Jan 6;6(1):e00738-20. doi: 10.1128/mSphere.00738-20 (PMC7845601; doi:10.1128/mSphere.00738-20)

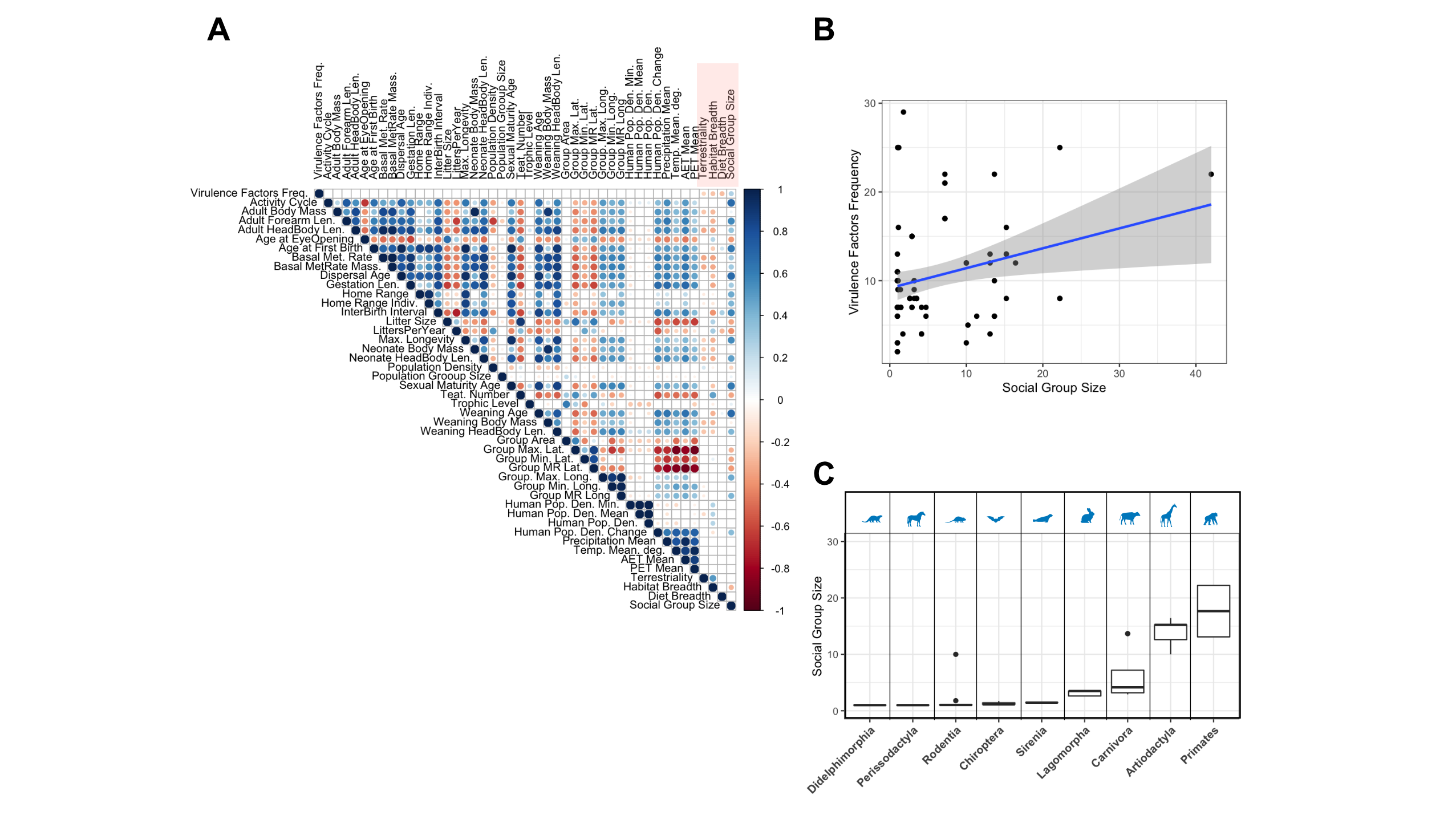

Supplement: FIG S4 [file mSphere.00738-20-sf004.tif]

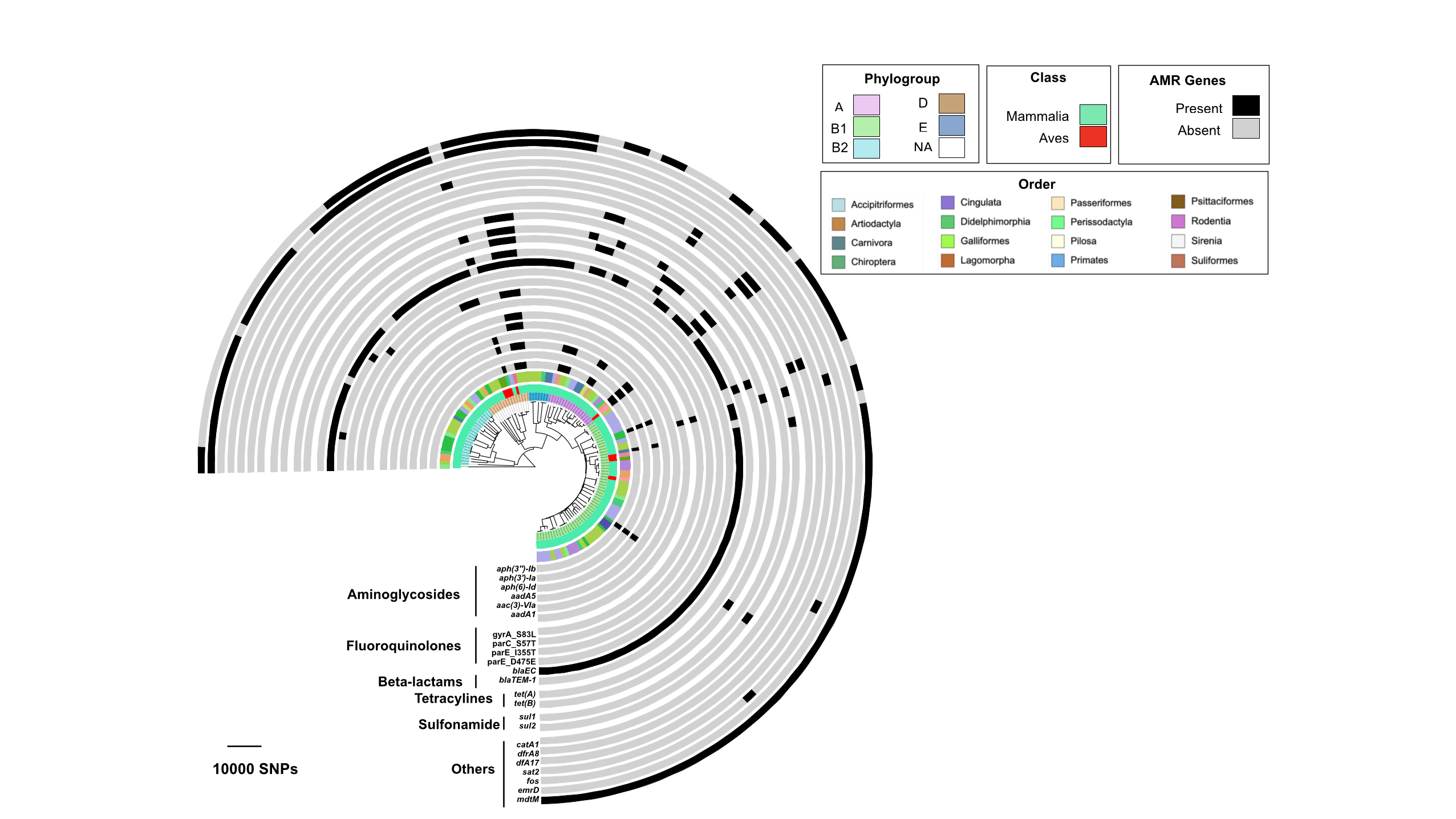

Supplement: FIG S5 [file mSphere.00738-20-sf005.tif]
